# Secret disclosure and social relationships in groups

**DOI:** 10.1177/13684302231187870

**Published:** 2023-08-21

**Authors:** Giovanni A. Travaglino, John M. Levine, Dominik-Borna Ćepulić, Zhuo Li

**Affiliations:** 1 Royal Holloway, University of London, UK; 2 University of Pittsburgh, USA; 3 Catholic University of Croatia, Croatia; 4 University of Western Ontario, Canada

**Keywords:** group dynamics, identity fusion, Relational Models theory, secrets

## Abstract

Personal secrets are a ubiquitous fact of group life, but the conditions under which they are revealed have not been explored. In five studies, we assessed secret disclosure in groups governed by four models of human sociality (Communal Sharing, Equality Matching, Authority Ranking, Market Pricing; Fiske). In Studies 1a and 1b, participants indicated their willingness to disclose secrets in hypothetical groups governed by the models. In Studies 2a and 2b, participants rated how much a group in which they disclosed secrets or nonsecrets is governed by the models. In Study 3, participants indicated their disclosure of various types of secrets in Communal Sharing and Equality Matching groups to which they belonged. Across studies, disclosure was most strongly associated with Communal Sharing, followed by Equality Matching. Study 3 further showed that identity fusion predicted disclosure in these two kinds of groups. Implications for understanding disclosure of personal secrets in group contexts were discussed.

Groups are critical building blocks of all human societies, and the study of groups has traditionally been a core area of social psychological inquiry. An understudied but important feature of groups is secrecy (Simmel, 1906/1950). Extending Simmel’s and other sociological analyses, Costas and Grey (2014) defined secrecy as “the ongoing formal and informal social processes of intentional concealment of information from actors by actors” (p. 426). Intentionality is a critical component of this definition, because unrevealed information that cannot be expressed (because it has been forgotten or is cognitively inaccessible for some other reason) is generally not viewed as secret (see also Bok, 1981).

Two forms of secrecy in groups can be differentiated. One form involves *collective* secrets, in which members withhold information *about the group* from others in *different* groups. An example is the mafia’s strict code of omertà, or silence (Paoli, 2003; Travaglino et al., 2016). A second form, which is the focus of the present article, involves *personal* secrets, in which members withhold information *about themselves* from others in the *same* group. Both kinds of secrets can have important implications for the group’s stability and capacity to achieve its objectives (cf. Fine & Holyfield, 1996).

While researchers have studied disclosure of personal secrets (e.g., Kelly, 1999; Slepian & Kirby, 2018; Slepian et al., 2017), very little effort has been directed toward understanding such disclosure in group contexts. Moreover, because the small amount of relevant work has focused on particular types of groups (e.g., families; Vangelisti, 1994), its generalizability is open to question. Finally, no theoretical framework has been offered to explain how features of groups influence their members’ tendency to disclose personal secrets. In the present studies, we drew on Fiske’s (2004) comprehensive theory of human sociality to derive and test hypotheses about how specific patterns of relationships among group members influence secret disclosure.

## Prior Work on Personal Secrets

Much of the work on personal secrets focuses on the intrapersonal and interpersonal costs of concealing information from others (e.g., W. A. Afifi & Afifi, 2020; Barasch, 2020; Baum & Critcher, 2020). The former costs derive from the fact that withholding information requires prospectively monitoring one’s speech in order to inhibit expressing cues that might reveal the secret (Critcher & Ferguson, 2014; see also McDonald et al., 2020; Slepian et al., 2015, 2016). This monitoring, in turn, increases stress and produces a number of undesirable cognitive, affective, and behavioral outcomes (Pachankis, 2007; Quinn & Chaudoir, 2009). Interestingly, these effects can be elicited by the mere *intention* to conceal information (Slepian et al., 2017, 2019, 2020), which produces mind-wandering and reduces well-being (Slepian et al., 2017). The interpersonal costs of concealing personal information can also be substantial. Withholding such information has been found to undermine the relationship between the secret keeper and others by reducing their relationship satisfaction (e.g., Finkenauer & Hazam, 2000), perceived closeness (e.g., Dailey & Palomares, 2004), and trust (e.g., Uysal et al., 2012).

Other work focuses on the benefits of revealing personal information. These include enhancing group members’ feeling of community by delineating boundaries between insiders and outsiders, increasing group cohesiveness, and strengthening members’ identification with the group (Bok, 1981; Goffman, 1959; Richardson, 1988; Simmel, 1906/1950; see Costas & Grey, 2014). Identification is particularly important, because it enhances members’ self-esteem and reduces their uncertainty (Ellemers & Haslam, 2011; Hogg, 2012). For instance, Paoli (2003) argued that, even before their status was illegal, mafia associations imported secrecy practices from Freemasons and other secret societies to increase members’ sense of community.

Revealing personal information is not always beneficial, of course, and hence people often hide such information because they fear its content will undermine their relationships. The potential risks as well as benefits of disclosure have stimulated several models of strategic self-presentation designed to clarify when people reveal personal information to others (e.g., T. Afifi & Steuber, 2009; Derlega & Grzelak, 1979; Jones & Pittman, 1982; Omarzu, 2000). For example, according to T. Afifi and Steuber’s (2009) Revelation Risk Model, people are more willing to reveal secrets when they desire catharsis, believe the target has the need or right to know the information, and assume the target wants them to reveal this information.

## Relational Models and Disclosure of Personal Secrets

Although our work, like that on the strategic self-presentation, focuses on the conditions under which people reveal personal information, it differs from prior analyses by examining the group characteristics associated with members’ willingness to reveal personal secrets to one another. Our research is based on Fiske’s (1992, 2004) Relational Models theory, which is arguably the most comprehensive and well-supported theory of human sociality. A major strength of the theory for our purposes is its applicability to groups varying widely on such dimensions as size, longevity, and goals. Although the theory has been fruitfully applied to many phenomena, including social cognition, emotions, psychopathology, sociolinguistics, moral judgment, and group processes, it has not been used to analyze the disclosure of secrets.

Relational Models theory posits that all forms of human interaction can be categorized according to four universal models. In *Communal Sharing* relationships, people are perceived and treated as equivalent and undifferentiated. In *Equality Matching* relationships, people seek to participate in balanced, one-for-one exchanges. In *Authority Ranking* relationships, individuals’ relative status determines their rights and obligations. And, in *Market Pricing* relationships, people desire socially meaningful ratios of some resource or outcome. Thus, the four relational models constitute distinct schemata regulating social interactions among group members (Fiske, 1992, 2004; Haslam & Fiske, 1999).

The four models have been found to influence a wide range of group phenomena, including distributive justice, decision-making, social influence, social identity, moral judgment, and aggression (see Haslam, 2004, for an overview). For example, regarding distributive justice, in Communal Sharing groups, members have equal access to common resources with no accounting of withdrawals; in Equality Matching groups, members receive identical shares of resources; in Authority Ranking groups, higher-ranked members have greater access to resources than do lower ranked members; and in Market Pricing groups, members receive shares proportional to some standard. Although group members’ interactions may be influenced by more than one relational model, a single model often dominates (cf. Lickel et al., 2001, 2006; see also Fiske, 1992).

Not surprisingly, given its scope, Relational Models theory shares some features with other theories of social interaction. However, no other theory provides as fine-grained an analysis or seeks to explain as broad a range of phenomena. For example, whereas Clark and Mills’s (2012) definition of communal relationships has much in common with Fiske’s definition of Communal Sharing relationships, their definition of exchange relationships combines elements of his Equality Matching and Market Pricing relationships, and they do not posit a parallel to his Authority Ranking relationships.

## The Present Studies

We conducted five studies examining the link between the relational model governing group members’ interactions and their willingness to disclose secrets to one another. We hypothesized that Communal Sharing groups would exhibit the strongest tendency to reveal secrets, compared to Equality Matching, Authority Ranking, and Market Pricing groups. This prediction was based on the assumption that the functions of revealing secrets, namely sharpened group boundaries, increased group cohesiveness, and enhanced group identification, are uniquely important in Communal Sharing groups, whose core defining features include the desire for similarity, consensus, solidarity, and clarity regarding who does and does not belong to the group (Fiske, 1992, 2004). Because disclosing secrets is potentially threatening, these features, which provide a strong sense of security, are likely to be particularly important (cf. Cozby, 1972).

We predicted that Equality Matching groups would exhibit the second strongest tendency to disclose secrets. These groups operate according to balanced, reciprocal exchanges in which members receive identical shares, make identical contributions, and have equal status (Fiske, 1992, 2004). Because revealed secrets may represent a form of reciprocal exchange, we expected these secrets to be relatively common in Equality Matching groups. This hypothesis is consistent with evidence that Equality Matching groups have higher perceived entitativity than do Market Pricing groups (Lickel et al., 2006) and also with research indicating that reciprocal self-disclosure in interpersonal relationships is associated with stronger closeness to the partner (e.g., Sprecher et al., 2013). We did not make differential predictions regarding secret disclosure in Authority Ranking and Market Pricing groups. In sum, we predicted that the four types of groups would be rank-ordered as follows in terms of secret disclosure: Communal Sharing > Equality Matching > Authority Ranking and Market Pricing.

## Studies 1a and 1b

### Goal

The goal of these studies was to test our hypotheses about the link between the relational model governing group members’ interactions and their willingness to reveal secrets to one another. Based on the reasoning presented above, we predicted that willingness to reveal secrets would be higher (a) in Communal Sharing groups than in the three other kinds of groups and (b) in Equality Matching groups than in Authority Ranking or Market Pricing groups. Study 1a tested our hypotheses using participants from western Europe. To assess the generalizability of our findings to a different cultural context, Study 1b replicated Study 1a using participants from southern China.

### Method

#### Participants

One hundred British participants (75 females, 25 males; M_age_ = 35.81, *SD* = 0.44) took part in Study 1a. We predetermined a sample size of 100 because this enabled us to detect a small-to-medium effect size, *f* = .12, at 80% power (α = .05), according to the G*Power software (v. 3.1.9.2; Faul et al., 2007). Participants were recruited using the Qualtrics software via the Prolific website and were paid a small monetary compensation for their participation. Sixty-eight participants from southern China took part in Study 1b as a classroom exercise (59 females, 9 males; *M_age_* = 20.53, *SD* = 0.66). Sample size depended on the availability of participants in the classroom during data collection. This sample size enabled us to detect a small-to-medium effect size, *f* = .14, at 80% power (α = .05).

#### Procedure

Participants in Study 1a were first asked to provide demographic information (i.e., gender and age). Next, they read a brief introduction to the study stating, “In this study, we are interested in understanding in which types of groups people are more likely to reveal personal secrets to other members. By personal secrets, we mean information about yourself, activities you take part in or relationships that you intentionally conceal from others.”

Participants were then provided with descriptions of four hypothetical groups in which members’ relationships were governed by one of the four relational models specified in Fiske’s (1992, 2004) theory (cf. Lickel et al., 2006). Each group was described using six descriptors adapted from Haslam’s (1994) Relational Models Scale. Example descriptors for each model included: “In this group, members would ‘give the shirt off their back’ for each other” (Communal Sharing); “If one of the group members does something for the other, the other tries to do the same thing in return next time” (Equality Matching); “In this group, one of the members takes most of the initiatives” (Authority Ranking); and “In this group, members act towards each other in a business-like way” (Market Pricing). The presentation order of the four group descriptions was randomized for each participant.

After reading each group description, participants were asked to answer three questions: “How likely you are to reveal a personal secret within this group?” (1 = *extremely unlikely*, 7 = *extremely likely*); “I would be willing to reveal a personal secret to other members of this group” (1 = *strongly disagree*, 7 = *strongly agree*); and “The nature of this group’s relationships would make it easier for me to reveal a personal secret” (1 = *strongly disagree*, 7 = *strongly agree*). Items formed reliable scales for each of the four descriptions (all αs > .92) and hence were averaged to produce a single score for each description.^1^

Study 1b used the same within-participants methodology as Study 1a, except that participants completed pencil and paper questionnaires. Materials were translated from English to Mandarin and then back translated to ensure accuracy. Again, items formed reliable scales for each of the four descriptions (all αs > .85) and hence were averaged to produce a single score for each description. On completion of the study, participants were thanked for their participation and debriefed.^2^

### Results

In both studies, data were analyzed using a one-way ANOVA with group type (Communal Sharing, Equality Matching, Authority Ranking, Market Pricing) as a within-participants factor. In Study 1a, the Mauchly’s test of sphericity was significant, *W* = .65, χ^2^(5, *N* = 100) = 42.42, *p* < .001, indicating a potential violation of the compound symmetry assumption. Thus, the Greenhouse-Geisser correction was applied to the standard *F* (Tabachnick & Fidell, 2014) in this study. The Mauchly’s test was not significant in Study 1b (*W* = .88, χ^2^(5, *N* = 68) = 8.09, *p* = .151).

#### Study 1a

Results indicated a significant main effect of group type, *F*(2.32, 229.56) = 100.54, *p* < .001, η_p_^2^ = .50. Participants were significantly more willing to reveal secrets in the Communal Sharing group (*M* = 5.30, *SD* = 1.53) than in the Equality Matching group (*M* = 4.08, *SD* = 1.45), the Authority Ranking group (*M* = 3.09, *SD* = 1.43), or the Market Pricing group (*M* = 2.49, *SD* = 1.39), *ps* < .001. In addition, willingness to reveal secrets was significantly higher in the Equality Matching group than in the Authority Ranking or Market Pricing group, *ps* < .001.

#### Study 1b

Results in this study also indicated a significant main effect of group type, *F*(3, 201) = 43.48, *p* < .001, η_p_^2^ = .40. Replicating the results of Study 1a, participants were significantly more willing to reveal secrets in the Communal Sharing group (*M* = 4.98, *SD* = 1.64) than in the Equality Matching group (*M* = 4.09, *SD* = 1.32), the Authority Ranking group (*M* = 3.10, *SD* = 1.25), or the Market Pricing group (*M* = 2.57, *SD* = 1.29), *ps* < .016. Moreover, willingness to reveal secrets was significantly higher in the Equality Matching group than in the Authority Ranking or Market Pricing group, *ps* < .001. Table 1 summarizes the confidence intervals for the mean comparisons for both studies.

**Table 1.** Confidence intervals for mean comparisons in Studies 1a and 1b.

		*Study 1a*		*Study 1b*	
Comparisons		95% Confidence interval for comparison			
		Lower limit	Upper limit	Lower limit	Upper limit
Communal Sharing	Equality Matching	0.93	1.45	0.40	1.39
	Authority Ranking	1.84	2.58	1.39	2.37
	Market Pricing	2.39	3.22	1.89	2.93
Equality Matching	Authority Ranking	0.65	1.34	0.59	1.40
	Market Pricing	1.25	1.94	1.12	1.92
Authority Ranking	Market Pricing	0.32	0.87	0.10	0.96

### Discussion

Taken together, the findings of these studies support our hypotheses about the link between the relational model governing group members’ interactions and their willingness to disclose secrets with one another. They also provide evidence for the generalizability of our findings from a western European to a southern Chinese context. In addition, though not predicted, participants in both studies were significantly more willing to reveal secrets in the Authority Ranking than in the Market Pricing group, *p_study1a_* < .001 and *p_study1b_* = .016.

## Studies 2a and 2b

### Goal

In Studies 1a and 1b, we asked participants how likely they were to reveal personal secrets in four hypothetical groups governed by different relational models (Fiske, 1992, 2004) and found that disclosing secrets was most likely in Communal Sharing groups, followed by Equality Matching groups. The findings demonstrated, for the first time, an association between a group’s relational model and its members’ willingness to reveal secrets to one another. Although the consistency of results across the two studies was reassuring, the methodology we employed posed three risks to the external validity of our findings. First, the gender composition of the two samples was skewed toward female participants. Second, participants were not asked about their propensity to reveal secrets to members of a group to which they belonged. Third, each hypothetical group was described as governed by a single relational model. As Fiske (1992, 2004) noted, however, group members’ interactions are sometimes governed by more than one model. For instance, two friends can have exchanges based on both the Communal Sharing model (e.g., sharing food equally) and the Market Pricing model (e.g., one person buying the other’s car at market price).

To address these limitations, Studies 2a and 2b employed a different methodology. In addition to recruiting samples with an approximately equal gender composition, we asked participants (a) to think of a group to which they belong and in which they reveal secrets and (b) to rate how much the group is governed by each of the four relational models. This latter feature allows the possibility that members’ interactions are governed by more than one model.

The present studies also extended Studies 1a and 1b in another way. Given our interest in the disclosure of personal secrets, participants in Studies 1a and 1b were asked how likely they were to reveal “information about yourself, activities you take part in or relationships that you intentionally conceal from others.” However, these instructions introduced an interpretive ambiguity, namely whether revealing *secret* information (activities . . . or relationships that you *intentionally conceal* . . .) is a necessary condition for our findings. Therefore, Studies 2a and 2b investigated the disclosure of nonsecret as well as secret information to other group members. The experimental design was thus a 2 (Type of Information: Personal Secret vs. Personal Nonsecret) × 4 (Relational Model: Communal Sharing, Equality Matching, Authority Ranking, Market Pricing) mixed model ANOVA with repeated measure on the Relational Model factor.

We expected results parallel to our earlier findings in the secret information condition. More specifically, we predicted that participants instructed to think about a group in which they revealed secrets would perceive the group as exemplifying Communal Sharing characteristics to a greater extent than Equality Matching characteristics, and Equality Matching characteristics to a greater extent than Authority Ranking and Market Pricing characteristics.

We expected a different pattern of results in the nonsecret information condition. Because revealing nonsecrets is typically less threatening than revealing secrets (e.g., since nonsecrets do not involve potentially stigmatizing information), people revealing nonsecrets may be relatively insensitive to the social context in which the disclosure occurs. Hence, we expected little variation in participants’ perceptions of the four relational models in the nonsecret information condition.

### Method

#### Participants

Two hundred and one British participants took part in Study 2a (101 females, 100 males; *M_age_* = 36.71, *SD* = 13.01; the extra female participant was oversampled by the platform). Two hundred British participants (102 females, 98 males; *M_age_* = 36.11, *SD* = 12.57) took part in Study 2b. In both studies, the sample size was predetermined at 200 participants, which enabled us to detect a small effect size, *f* = .08, at 80% power (α = .05). Participants in both studies were recruited online using Qualtrics software and the platform Prolific Academic. Participants were randomly assigned to the two Type of Information conditions.

#### Procedure

After providing demographic information (i.e., gender and age), participants in Study 2a read the following: “During their lives, individuals participate in many groups. By ‘groups,’ we mean three or more individuals who share common characteristics and/or interact together to achieve goals. Please, think about a group to which you belong.”

The remaining instructions varied depending on Type of Information condition. Participants in the Personal Secret condition read, “Specifically, we would like you to think about a group in which you share personal secrets with other members. By personal secrets, we mean information about yourself, activities you take part in, or relationships that you intentionally conceal from others.” In contrast, participants in the Personal Nonsecret condition read, “Specifically, we would like you to think about a group in which you share personal information with other members. By personal information, we mean information about yourself, activities you take part in, or relationships that you have, and that you have no issues telling others about.”

Following the instructions, participants were asked to rate the group they were thinking of on 24 items measuring the four relational models (Haslam, 1994). Each relational model was measured by six items. Sample items included: “‘What’s mine is yours’ is true of the relationship between people in the group” and “‘One for all and all for one’ is true of the relationship within this group” (Communal Sharing, α = .88); “If a member of the group shares something with another person in the group they would divide it down the middle” and “In this group, people are pretty equal in the things they do for each other” (Equality Matching, α = .76); “One person in the group tends to lead” and “One person in the group takes most responsibility” (Authority Ranking, α = .66); and “People in the group act towards others in the group in a purely rational way” and “People in the group choose to participate in this relationship when it is worth their while to do so” (Market Pricing, α = .89). On completion of the study, participants were thanked, paid a small monetary compensation, and debriefed.

The procedure in Study 2b was identical to that in Study 2a, except for the following changes. First, in order to increase the salience of group they were thinking about, participants in Study 2b were asked to write down that group. Second, because the definition of nonsecret information in Study 2a may have been somewhat vague, this definition was sharpened in Study 2b. In the Personal Nonsecret condition of this study, participants read the following: “Please, think about a group to which you belong and within which you share many pieces of information about you with other members. By information about you, we mean information about yourself, activities you take part in, or relationships that many people outside this group already know about you, and that you have no reluctance telling others about.” Again, items formed reliable scales for the four descriptions (Communal Sharing, α = .87; Equality Matching, α = .76; Market Pricing, α = .72; Authority Ranking, α = .86) and were averaged to produce a single score for each description.

### Results

In both studies, data were analyzed using a 2 (Type of Information: Personal Secret vs. Personal Nonsecret) × 4 (Relational Model: Communal Sharing, Equality Matching, Authority Ranking, Market Pricing) mixed model ANOVA with repeated measure on the Relational Model factor. The Mauchly’s test of sphericity was significant in both Study 2a, *W* = .60, χ^2^(5, *N* = 201) = 100.79, *p* < .001, and Study 2b, *W* = .64, χ^2^(5, *N* = 190) = 82.82, *p* < .001. Therefore, the Greenhouse-Geisser correction was applied to the standard *F* in both studies. Ten participants were excluded from analyses in Study 2b because they did not indicate any group when asked to do so.

#### Study 2a

Analyses revealed a significant main effect of relational model, *F*(2.26, 450.53) = 37.74, *p* < .001, η_p_^2^ = .16, indicating that, across type of information, participants perceived their group as significantly higher on Communal Sharing characteristics (*M* = 4.60, *SD* = 1.36) than on Equality Matching (*M* = 4.38, *SD* = 1.12), Authority Ranking (*M* = 3.85, *SD* = 1.39), or Market Pricing (*M* = 3.53, *SD* = 0.95), *ps* < .015, characteristics. In addition, participants viewed their group as significantly higher on Equality Matching characteristics than on Authority Ranking or Market Pricing characteristics, *ps* < .001, and the Authority Ranking–Market Pricing difference was significant, *p* = .001. The main effect of type of information was not significant, *F*(1, 199) = 1.19, *p* = .276, η_p_^2^ = .006.

**Figure 1.**
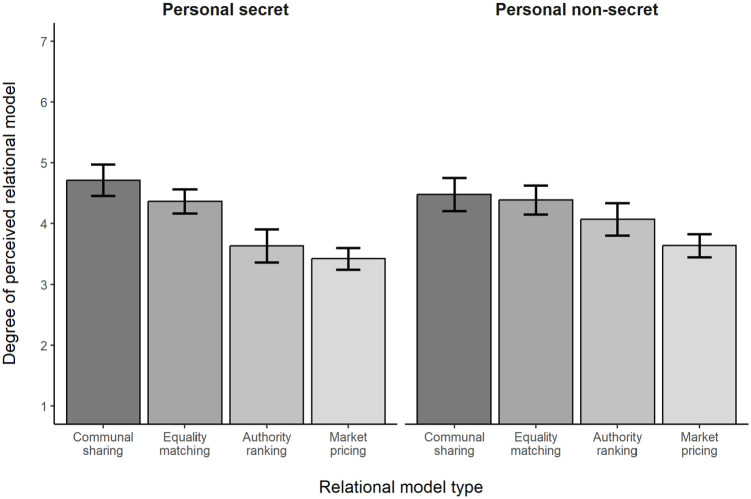
Degree of perceived relational model as a function of relational model type in Study 2a. *Note.* Bars show observed means and their 95% confidence intervals.

There was also a significant Type of Information × Relational Model interaction, *F*(2.26, 450.53) = 3.24, *p* = .034, η_p_^2^ = .02 (see Figure 1). Regarding the simple effects of relational model within the personal secret information condition, as predicted Communal Sharing (*M* = 4.71, *SD* = 1.32) was significantly higher than Equality Matching (*M* = 4.37, *SD* = 1.02), Authority Ranking (*M* = 3.6, *SD* = 1.40), and Market Pricing (*M* = 3.42, *SD* = 0.92), all *ps* ⩽ .007. Moreover, Equality Matching was significantly higher than Authority Ranking and Market Pricing (*ps* < .001), which did not differ significantly from one another (*p* = .112). The simple effects of relational model within the personal nonsecret information condition revealed a different pattern. Communal Sharing (*M* = 4.48, *SD* = 1.39) was significantly higher than Authority Ranking (*M* = 4.07, *SD* = 1.36, *p* = .034) and Market Pricing (*M* = 3.64, *SD* = 0.97; *p* < .001) but not Equality Matching (*M* = 4.39, *SD* = 1.21; *p* = .474). Equality Matching was also significantly higher than Market Pricing, *p* < .001, but not Authority Ranking, *p* = .068, and Authority Ranking and Market Pricing differed significantly, *p* = .001. Table 2 summarizes the confidence intervals for the mean comparisons.

**Table 2.** 95% confidence intervals for mean comparisons in Study 2a and 2b.

Condition			*Study 2a*		*Study 2b*	
			Lower limit	Upper limit	Lower limit	Upper limit
Personal secrets	Communal Sharing	Equality Matching	0.10	0.60	0.04	0.51
		Authority Ranking	0.71	1.46	0.71	1.42
		Market Pricing	0.94	1.64	1.04	1.72
	Equality Matching	Authority Ranking	0.39	1.08	0.44	1.13
		Market Pricing	0.68	1.21	0.82	1.40
	Authority Ranking	Market Pricing	-0.47	0.05	-0.58	-0.06
Personal information	Communal Sharing	Equality Matching	-0.16	0.34	-0.05	0.42
		Authority Ranking	0.03	0.79	0.24	0.95
		Market Pricing	0.49	1.19	0.48	1.17
	Equality Matching	Authority Ranking	-0.02	0.66	0.06	0.75
		Market Pricing	0.48	1.02	0.35	0.92
	Authority Ranking	Market Pricing	0.17	0.69	-0.49	0.03

#### Study 2b

Analyses again indicated a significant effect of relational model, *F*(2.33, 437.42) = 43.30, *p* < .001, η_p_^2^ = .19, indicating that, across types of information, participants perceived their group as significantly higher on Communal Sharing characteristics (*M* = 4.84, *SD* = 1.24) than on Equality Matching (*M* = 4.61, *SD* = 1.05), Authority Ranking (*M* = 3.91, *SD* = 1.26), or Market Pricing (*M* = 3.60, *SD* = 1.04), *ps* < .007, characteristics. Equality Matching was also significantly different from Authority Ranking and Market Pricing, *ps* < .001, and the latter two models differed significantly from each other, *p* = .004. The main effect of type of information was not significant, *F*(1, 188) = 0.01, *p* = .930, η_p_^2^ < .01.

**Figure 2.**
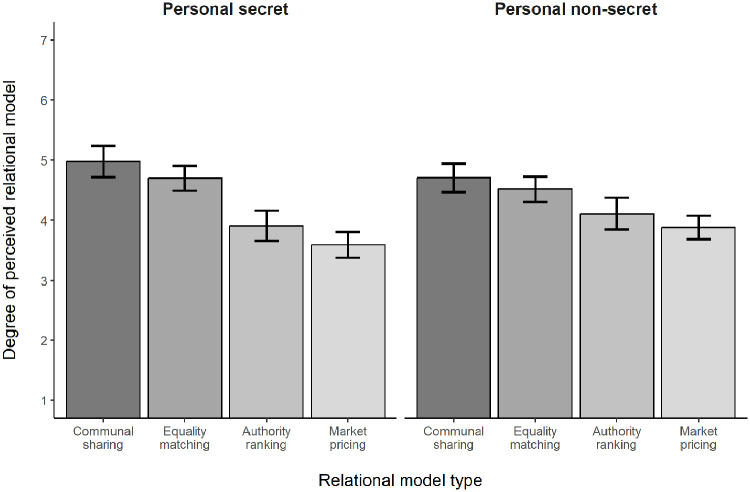
Degree of perceived relational model as a function of relational model type in Study 2b. *Note.* Bars show observed means and their 95% confidence intervals.

The Relational Model × Type of Information interaction was also significant, *F*(2.33, 437.42) = 3.15, *p* = .036, η_p_^2^ = .02 (see Figure 2). Regarding the simple effects of relational model within the personal secret information condition, as predicted Communal Sharing (*M* = 4.98, *SD* = 1.30) was significantly higher than Equality Matching (*M* = 4.70, *SD* = 1.04), Authority Ranking (*M* = 3.91, *SD* = 1.26), and Market Pricing (*M* = 3.60, *SD* = 1.07), all *ps* ⩽ .022. Equality Matching was significantly higher than Authority Ranking and Market Pricing, *ps* < .001. The latter two models also differed significantly from each other, *p* = .016. Thus, findings in the personal secret information condition (Communal Sharing > Equality Matching > Authority Ranking and Market Pricing) were consistent with those in Studies 1a, 1b, and 2a.

The simple effects of relational model within the personal nonsecret information condition revealed that Communal Sharing (*M* = 4.71, *SD* = 1.17) was significantly higher than Authority Ranking (*M* = 4.11, *SD* = 1.31, *p* = .001) and Market Pricing (*M* = 3.88, *SD* = 0.99; *p* < .001) but not Equality Matching (*M* = 4.51, *SD* = 1.05; *p* = .118). Equality Matching was also significantly higher than Authority Ranking and Market Pricing (*ps* ⩽ .020), and Authority Ranking and Market Pricing did not differ significantly (*p* = .085). Table 2 summarizes the confidence intervals for the mean comparisons.

These findings are quite similar to those in Study 2a, demonstrating again that the social context in which disclosure occurs matters even when the threat value of the disclosed information is low. In addition, whereas participants in the personal secret information condition perceived their group as significantly higher on Communal Sharing than Equality Matching characteristics, those in the personal nonsecret information condition did not.

### Discussion

As predicted, results in the personal secret information condition of Studies 2a and 2b paralleled those in Studies 1a and 1b (Communal Sharing > Equality Matching > Authority Ranking and Market Pricing). It is noteworthy that these findings were obtained using a very different paradigm than employed in the earlier studies. We also found, contrary to expectations, that participants’ perceptions of the four relational models varied in the personal nonsecret information condition, indicating that the social context in which disclosure occurs mattered even when the threat value of the disclosed information was low.

An interesting difference between the secret and nonsecret information conditions is worth pointing out. Whereas participants in the former condition perceived their group as significantly higher in Communal Sharing than Equality Matching characteristics, this was not true in the latter condition. Across studies, results in the secret information condition are consistent with our initial assumption that secrets are more likely to be shared in Communal Sharing groups than exchanged in Equality Matching groups, because the functions of disclosing secrets—sharpened group boundaries and enhanced group identification—are very important to Communal Sharing groups. Because these functions are less relevant to disclosing nonsecrets, it is not surprising that participants did not perceive their group as higher in Communal Sharing than Equality Matching characteristics.

## Study 3

### Goal

All of the previous studies obtained evidence indicating more disclosure of personal secrets in Communal Sharing than in Equality Matching groups. One goal of Study 3 was to test the generalizability of this difference using a variant of the methodology employed in Studies 1a and 1b, in which participants were provided with descriptions of hypothetical groups exemplifying each of the four relational models specified in Fiske’s (1992) theory and then asked how likely they were to reveal an unspecified personal secret to members of each group. As in Studies 1a and 1b, participants in the current study were presented with lists of features describing Communal Sharing and Equality Matching relational models.^3^ However, unlike Studies 1a and 1b, they were asked (a) to write down a group to which they belonged that matched the features of each kind of group and (b) to list the number of members of each group to whom they disclosed several types of personal secrets. Thus, participants’ responses were based on actual, rather than hypothetical, group memberships. Based on our earlier findings, we predicted that participants would report disclosing secrets to more members of their Communal Sharing group than their Equality Matching group.

A second goal of the current study was to investigate two potential predictors of secret disclosure in Communal Sharing and Equality Matching groups—identity fusion in Communal Sharing groups and the norm of reciprocity in Equality Matching groups.

According to Rai and Fiske (2011), social relations within Communal Sharing and Equality Matching groups are based on different moral motives, namely unity and equality respectively. In the case of Communal Sharing groups, “Unity is directed toward caring for and supporting the integrity of in-groups through a sense of collective responsibility and common fate. . . . Unity often facilitates intense care and sacrifice for those within the Communal Sharing relation” (pp. 61–62). These characteristics mirror those of identity fusion, defined as “a visceral sense of oneness with the group,” which strengthens the relationships among members and their willingness to sacrifice for the group (e.g., Swann & Buhrmester, 2015; Swann et al., 2014; see also Thomsen & Fiske, 2018), Therefore, we expected to find an interaction between identity fusion and group type, such that identity fusion would be positively associated with willingness to reveal secrets in Communal Sharing but not in Equality Matching groups.

In the case of Equality Matching groups, “Equality is directed toward enforcing even balance and in-kind reciprocity in social relations . . . Equality provides the moral motivation for maintaining ‘scratch my back and I will scratch yours’ forms of reciprocity and pursuing eye-for-an-eye forms of revenge” (Rai & Fiske, 2011, p. 63). Previous research indicates that reciprocal exchanges of information in interpersonal relationships strengthen social bonds (Sprecher et al., 2013). Therefore, we expected an interaction between reciprocity and group type, such that the perceived strength of the reciprocity norm would be positively associated with willingness to reveal secrets in Equality Matching but not in Communal Sharing groups.

### Method

#### Participants

Two hundred and fifty British participants took part in the study (124 females, 114 males, 12 participants did not indicate their gender; *M_age_* = 30.61, *SD* = 10.99). The sample was predetermined at 250 participants, who were recruited online using Qualtrics software and the platform Prolific Academic. A power analysis was not conducted, because no software exists to perform this analysis for beta-binomial mixed models.

#### Procedure

Participants first read the following brief introduction: “In this study, we are interested in understanding when people reveal personal secrets to other members of their formal or informal groups.” Participants were then provided with the same definitions of “groups” and “personal secrets” as in our previous studies. Next, participants were presented with two lists of six features describing either a Communal Sharing or an Equality Matching relational model. (The order of presentation of the lists was randomized across participants.) These features were the same as those used in Studies 1a and 1b (e.g., Communal Sharing: “What happens to members of this group is almost as important to other members as what happens to them”; Equality Matching: “Group members more or less keep track of favors and obligations”).

After reading each list, participants were asked to write down a group to which they belonged in which members’ relationships matched the features listed. They then answered several questions related to that group (see below). Participants next completed the same procedure for the second group (either Communal Sharing or Equality Matching, depending on the order in which the groups were presented).

For each relational model group, participants were asked to indicate the total number of people in the group, their level of identity fusion with the group, and the strength of the reciprocity norm in the group.^4^ Level of identity fusion was measured using the 7-item scale developed by Gómez et al. (2011; Communal Sharing, α = .79; Equality Matching, α = .85). Sample items are “I am one with my group,” and “I feel immersed in my group.” The strength of the norm of reciprocity was measured using four items: “If I help other group members, they help me in return,” “I feel obligated to help others who help me,” “Members who don’t reciprocate favors are violating group norms,” “The rule in this group is, I won’t help you unless you help me.” However, responses to these items were only weakly correlated (Communal Sharing, *r*_average_ < .22; Equality Matching, *r*_average_ < .12), and the items did not form a reliable scale (Communal Sharing, α = .54; Equality Matching, α = .34). Therefore, we included the four items separately in our model to explore their effects.

Next, participants were presented with a list of 13 types of secrets drawn from a larger typology developed by Slepian et al. (2017). Examples included: “Dislike a friend, or are unhappy with your current social life,” “Used illegal drugs, or abused/addicted to a legal drug (e.g., alcohol, painkillers),” and “Thought about having sexual relations with another person (while already in a relationship)” (see Codebook, Study 3 for the full list). These secrets were selected because they are among the most frequently confided secrets reported in Slepian and Greenaway (2018) and involve a diverse range of topics.^5^ For each secret, participants were asked to report the number of group members to whom they had told the secret. (If participants had not told the secret to anyone in the group, they were asked to write down zero.) Finally, participants completed demographic measures (gender and age), were debriefed, and were compensated for their time.

### Results

Twenty-five participants were excluded from the analyses because they failed to write down a group, reported a group smaller than three members (which violated instructions) or larger than 100 members (which occurred very rarely), or indicated the same group for both types of relational models.

In analyzing the impact of group type, identity fusion, reciprocity-related items, and secret types on the number of group members with whom participants shared secrets, we used the R-packages tidyverse (Wickham et al., 2019), glmmTMB (Brooks et al., 2017), emmeans (Lenth, 2020), performance (Lüdecke et al., 2020) and ggeffects (Lüdecke, 2018). We fitted a generalized linear mixed model for a binomial response variable (estimated using maximum likelihood and nlminb optimizer). Group type, identity fusion, and reciprocity-related items were treated as fixed effects, as were the interactions of group type with identity fusion and reciprocity-related items. Participants and secret types were treated as random effects. Predictors in the model were centered. The total number of members in each group were used as weights in the binomial mixed-effects regression.

Comparison of the observed versus simulated model residuals (*n* = 500 simulations) using the DHARMa package (Hartig, 2020) indicated that zero-inflation occurred. Furthermore, the ratio of sum of squared Pearson residuals and the residual degrees of freedom (*χ*^2^ = 20582.52, *df_resid_* = 5783, ratio = 3.56, *p* < .001) calculated by the function *overdisp_fun*, provided by Bolker (2020), suggested that the model exhibited overdispersion. Because overdispersion can lead to biased parameter estimates and standard errors (Harrison, 2014, 2015; Hilbe, 2011), we estimated the parameters using the beta-binomial model, which accounts for overdispersion through an additional constant overdispersion term *Φ* (for details, see Harrison, 2015; Richards, 2008). Although there was still some deviation from the expected distribution of residuals (mainly for extreme values of predictors where fewer data points were present), the DHARMa diagnostics indicated that the beta-binomial model is more adequate than the original binomial one.

We first tested a model including all four reciprocity-related items and identity fusion. Two items (“Members who don’t reciprocate favors. . .”; “The rule in this group is. . .”) interacted significantly with Group Type. Responses on the former item were positively associated with revealing secrets in Communal Sharing groups, whereas responses on the latter item were negatively associated with revealing secrets in these groups. Moreover, none of the four items was significantly associated with secret disclosure in Equality Matching groups. In light of these weak and inconsistent findings, we dropped the four reciprocity-related items from our predictive model. Doing so did not impact the significance of the other predictors in the model. (The initial model including the four reciprocity-related items is reported in the Supplemental Material, Table A and Figures A–B.)

Results for the final model are summarized in Table 3. Although the explanatory power of the model was not high (see the Nakagawa’s *R*^2^_*marginal*_ / *R*^2^_*conditional*_ in Table 3),^6^ consistent with our prediction and previous findings, participants revealed secrets to a larger proportion of members in Communal Sharing groups than in Equality Matching groups, *b* = −0.24, *p* < .001. Specifically, after fixing identification at its mean, the model indicated that participants disclosed their secrets to 18.00% of members in Communal Sharing groups and 15.00% in Equality Matching groups.

**Table 3.** Fixed and random effects of the generalized linear mixed model in Study 3.

Effect	Parameter	*b*	*SE*	95% CI	*t ratio*	*p*
	(Intercept)	-1.65	0.15	[-1.94, -1.35]	-10.89	< .001
Fixed	group [Equality Matching]	-0.24	0.05	[-0.35, -0.14]	-4.47	< .001
	(Intercept) [Communal Sharing]	-1.52	0.15	[-1.82, -1.22]	-9.93	< .001
	(Intercept) [Equality Matching]	-1.77	0.15	[-2.07, -1.47]	-11.52	< .001
	identity fusion	0.35	0.04	[0.27, 0.43]	8.75	< .001
	identity fusion × group [Equality Matching]	0.19	0.06	[0.07, 0.31]	3.16	.002
	identity fusion [Communal Sharing]	0.25	0.05	[0.15, 0.36]	4.76	< .001
	identity fusion [Equality Matching]	0.44	0.05	[0.35, 0.54]	9.54	< .001
Random	*Var*(secret type)	0.23				
	*Var*(subject)	1.00				
Nakagawa’s *R*^2^*_marginal_ / R*^2^_*conditional*_		0.06 / 0.39^a^				

*Note.* Overdispersion parameter for the beta-binomial family was 1.54. The outcome variable is expressed as the logit of proportion of group members to whom the secret was revealed. Indented entries show simple effects. Communal sharing is coded 0 and equality matching is coded 1 in the *group* variable.

a Because it was not possible to estimate the *R*^2^ values for the beta-binomial model using Lüdecke et al.’s (2020) package, we used estimates from the original binomial model.

**Figure 3.**
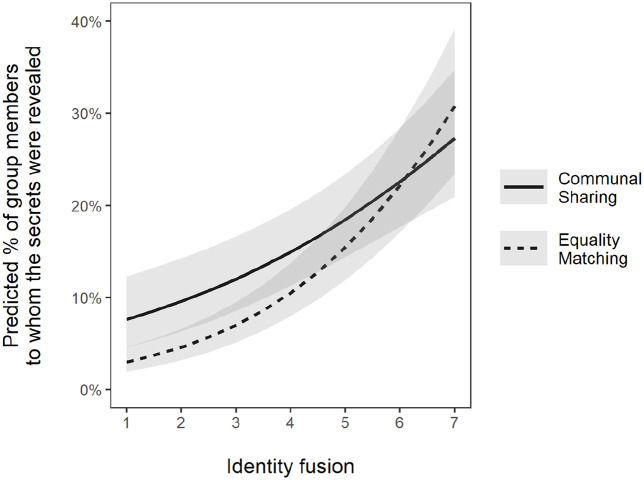
Marginal effects of identification in communal sharing (Communal Sharing) and equality matching (Equality Matching) groups in Study 3. *Note.* The grey area around lines represents the 95% confidence interval of the parameters.

As Figure 3 shows, identity fusion was positively associated with the proportion of members to whom participants disclosed secrets in *both* types of groups. In addition, a significant Group Type × Fusion interaction, *b* = 0.19, *p* = .002, revealed that the slope of this relationship was steeper in Equality Matching groups, *b* = 0.44, *p* < .001, than in Communal Sharing groups, *b* = 0.25, *p* < .001. This difference was due to lower predicted disclosure in Equality Matching than Communal Sharing groups at low levels of fusion. At high levels, disclosure was very similar in the two kinds of groups.

**Figure 4.**
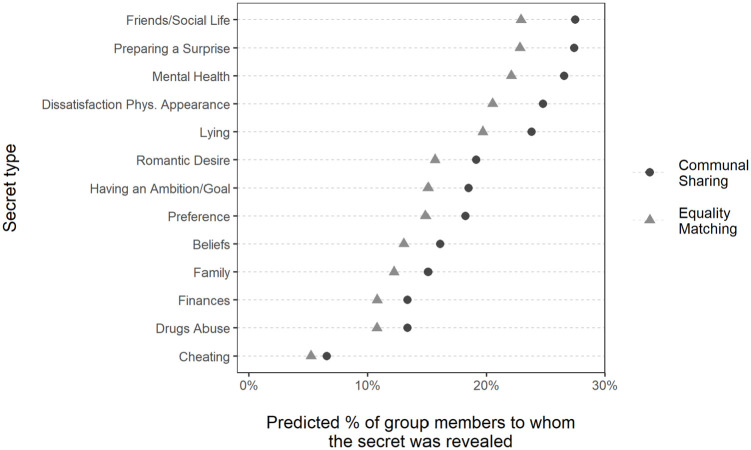
Distribution of random intercepts transformed to the predicted percentages of group members to whom different secret types were revealed in Study 3. *Note.* Reciprocity and identification are fixed at their means, whereas the subject-level random effects are held at their population level. The categories of Secrets were adapted from Slepian et al. (2017). *Friends/Social Life*: “Dislike a friend, or are unhappy with your current social life”; *Surprise*: “Planning a surprise for someone (other than a marriage proposal)”; *Mental Health*: “Had mental health issues, or are dissatisfied with something about yourself other than physical appearance (such as fears, anxieties, depression, mental disorders, eating disorders)”; *Dissatisfaction Phys. Appearance*: “Dissatisfied with your physical appearance”; *Lying*: “Have lied to someone”; *Romantic Desire*: “Had romantic desires about someone (while being single). For example, a crush, being in love with someone, wanting relations with a specific person (while being single)”; *Ambition/Goal*: “Kept a secret ambition, secret plan, or secret goal for yourself?”; *Preference*: “Kept secret a preference for something?”; *Beliefs*: “Kept a belief secret? (for example, political views, religious views, views about social groups, prejudice)”; *Family*: “Kept a detail about your family secret?” *Finances*: “Kept secret details about finances (or amount of money you have)?”; *Drug Abuse*: “Used illegal drugs, or abused/addicted to a legal drug (e.g., alcohol, painkillers)”; *Cheating*: “Thought about having sexual relations with another person (while already in a relationship).”

To assess participants’ tendency to reveal specific types of secrets, we examined the random intercepts for different secrets. As Figure 4 shows, secrets about Friends/Social Life, Surprise, and Mental Health were the most likely to be revealed, whereas secrets regarding Finances, Drug Abuse, and Cheating were the least likely to be revealed (see Figure 4 Note for question wording). (Figure C in the Supplemental Material shows the observed means.)

To assess whether disclosure of different secret types varied in Communal Sharing and Equality Matching groups, we examined the significance of the random effect reflecting differences in group type across secret types. In the first model we tested, we fixed the random effect (reflecting differences in group type across secret types) to be orthogonal to the random intercept. In the second model, we allowed the correlation between the random effect and intercept to be freely estimated. The variance of the added random effect in the first model was very close to zero, and the fit of this model was not significantly better than the fit of the base model (Δχ^2^(1) = .01,  *p* > .999). Similarly, the second model did not fit the data better than the base model (Δχ^2^(2) = 0.69,  *p* = .709), and it had convergence issues. These results indicate that, on the logit scale, participants’ greater propensity to reveal secrets in Communal Sharing than in Equality Matching groups was consistent across types of secrets.

### Discussion

We predicted and found that participants disclosed secrets to a larger proportion of members of Communal Sharing than of Equality Matching groups to which they belonged. These findings paralleled results from our previous studies, which were obtained using different methodologies and different operationalizations of secret disclosure. In addition, the present study indicated that participants’ tendency to reveal secrets to more members of their Communal Sharing than Equality Matching groups was not restricted to particular types of secrets but instead generalized across a broad range of secrets.

We also hypothesized that the level of identity fusion would be positively associated with secret disclosure in Communal Sharing (but not in Equality Matching) groups, whereas the perceived strength of the reciprocity norm would be positively associated with disclosure in Equality Matching (but not in Communal Sharing) groups. However, results revealed a different pattern.

We found that identity fusion was positively associated with secret disclosure in both Communal Sharing and Equality Matching groups, and the extent of disclosure was very similar across group types at high levels of fusion (Figure 3). That identity fusion enhanced secret disclosure in Equality Matching as well as Communal Sharing groups is interesting in light of the different moral motives assumed to underlie social relations in the two kinds of groups. Thus, rather than being important only in Communal Sharing groups based on unity, feelings of closeness to other members also influenced secret disclosure in Equality Matching groups based on equality/reciprocity.

Given these results, it is plausible that identity fusion may also influence disclosure in the other kinds of groups identified by Fiske (1992), namely Authority Ranking groups, based on the moral motive of hierarchy, and Market Pricing groups, based on proportionality (Rai & Fiske, 2011). Although our previous studies indicated relatively little disclosure of secrets in Authority Ranking and Market Pricing groups, it would be informative to investigate the relationship between fusion and disclosure in these groups.

Just as the relationship between identity fusion and secret disclosure was similar in Communal Sharing and Equality Matching groups, so the relationship between the strength of the reciprocity norm and disclosure was similar in these groups—with a major difference. Whereas identity fusion had a significant and consistent (positive) impact on revealing secrets in Communal Sharing and Equality Matching groups, the strength of the reciprocity norm (based on analyses of four items) had a weak and inconsistent impact in both kinds of groups. The reciprocity norm was not expected to influence disclosure of secrets in Communal Sharing groups, but its failure to do so in Equality Matching groups was surprising. Why might it have occurred?

One possibility is that the items we used to measure the reciprocity norm did not capture the essence of this construct. This interpretation is implausible in light of the strong face validity of these items (“If I help other group members, they help me in return,” “I feel obligated to help others who help me,” “Members who don’t reciprocate favors are violating group norms,” “The rule in this group is, I won’t help you unless you help me”). A second possibility is that, contrary to Fiske’s (1992, 2004; Rai & Fiske, 2011) Relational Models theory, the norm of reciprocity is not important in Equality Matching groups. This interpretation is not persuasive in light of the strong conceptual and empirical basis for the theory.

A third possibility is that, although reciprocity underlies important processes in Equality Matching groups (e.g., distributive justice, moral judgment), it does not influence the focal dependent variable in our studies—group members’ willingness to disclose personal secrets to fellow members. Instead, identify fusion was associated with secret disclosure in Equality Matching as well as Communal Sharing groups. Moreover, identity fusion’s predictive power remains constant regardless of whether reciprocity items are included in the model (see Supplemental Material, Figure B). Perhaps because secrets often contain potentially stigmatizing information, their disclosure is driven by feelings of closeness in most, if not all, kinds of groups.

## General Discussion

The present studies sought to clarify the link between the relational model governing group members’ interactions and their willingness to reveal personal secrets to other members. Our work was based on Fiske’s (1992, 2004) Relational Models theory, which identifies four universal models of sociality – Communal Sharing, Equality Matching, Authority Ranking, and Market Pricing. We expected the strongest tendency to share secrets within Communal Sharing groups, followed by Equality Matching groups. Five experiments were conducted.

In Studies 1a and 1b, participants read descriptions of hypothetical groups governed by Fiske’s four relational models and then indicated their willingness to disclose personal secrets within each group. Across both studies, groups were rank-ordered as follows: Communal Sharing > Equality Matching > Authority Ranking and Market Pricing. In Studies 2a and 2b, participants thought about a membership group in which they disclosed personal information and then rated how much the group was governed by each relational model. In addition, whether the disclosed information was secret or nonsecret was manipulated. In the secret condition, perceptions of group type were rank-ordered as follows: Communal Sharing > Equality Matching > Authority Ranking and Market Pricing. Results in the nonsecret condition were quite similar, except that the difference between Communal Sharing and Equality Matching groups was not significant. Finally, in Study 3, participants provided retrospective reports of secret disclosure in Communal Sharing and Equality Matching membership groups. Parallel to previous findings, disclosure was greater in Communal Sharing than Equality Matching groups, and this difference was robust across secret type. Moreover, identity fusion was positively associated with disclosure in both kinds of groups.

Taken as whole, our studies provide strong support for our hypotheses regarding the relationship between group members’ propensity to reveal secret information and the relational model governing group interactions. Moreover, because our studies were based on a conceptual framework (Fiske’s Relational Models theory) that is applicable to all types of groups and because our methodologies did not restrict the types of groups that participants responded to, our data have broad generalizability.

It is worth noting that our findings regarding disclosure of nonsecret information in Studies 2a and 2b suggest two modifications to our original thinking. First, they indicate that Fiske’s theory is relevant to the disclosure of personal information *in general*, not just secret information. Second, they suggest that group members’ propensity to disclose more personal information in Communal Sharing than Equality Matching groups applies primarily to secret information.

### Limitations and Future Directions

A limitation of Studies 1a and 2a, which used hypothetical groups, was eliminated in Studies 2a and 2b and Study 3, which used participants’ membership groups. In all cases, however, participants responded to cognitively represented groups. It would therefore be valuable to investigate the amount and character of secret disclosure in interacting groups governed by differing relational models.

Our studies also suggest additional questions for future research. For example, as we noted, Fiske (1992, 2004) argued that group members’ interactions are sometimes governed by more than one model. We attempted to address this feature of Relational Models theory in Studies 2a and 2b by asking participants to think of a group to which they belong and in which they reveal secrets and to rate how much the group is governed by each of the four relational models. Although the latter instructions allowed the possibility that members’ interactions were governed by more than one model, it would would be useful in future studies to focus more explicitly on how different combinations of relational models influence the disclosure of secrets.

It would also be informative to consider additional predictors of group members’ willingness to disclose personal secrets. One important predictor is likely to be a potential discloser’s feeling of closeness to a potential target of disclosure. This feeling may derive from two sources. One is the valence and intensity of the pre-existing personal relationship between the two individuals. The other is their shared social identity based on a common group membership. Disclosure is likely to be greater to liked vs. disliked members of one’s group. Moreover, disclosure is likely to be greater in groups with which one is strongly vs. weakly identified. Therefore, in order to understand secret disclosure in groups, it is important to understand how potential disclosers perceive and evaluate their group memberships.

Additional attention should also be given to how willingness to disclose secrets is influenced by the nature of the secrets. Although Study 3 revealed no significant differences across 13 types of secrets, these secrets were drawn from a typology developed in the context of interpersonal relationships (Slepian et al., 2017). Future research should develop a typology of secrets specific to group contexts and assess their impact on disclosure.

Yet another question for future research is the directionality of the association between the relational model(s) governing group members’ interactions and their propensity to reveal personal secrets. Although our studies suggest that certain features of relational models, in particular the level of identity fusion they elicit, can stimulate the disclosure of personal secrets, the reverse causal direction is also plausible. For example, sociologically-oriented theorists (e.g., Costas & Grey, 2014; Goffman, 1959) have suggested that disclosing secrets within a group can increase members’ identification with it. Bi-directional causal relationships are also possible, as when disclosing-induced identification stimulates members to reveal additional personal secrets.

Finally, potential relationships between group members’ disclosure of personal secrets to one another and their disclosure of group (collective) secrets to outgroup members deserve attention. Fiske’s Relational Models theory may also be relevant to these latter secrets. For example, members of Communal Sharing and Equality Matching groups characterized by high levels of identity fusion may be less likely to reveal collective secrets than may members of Authority Ranking and Market Pricing groups. This hypothesis, based on the assumption that group identification stimulates group loyalty (Levine & Moreland, 2002), is consistent with the extensive literature on identity fusion (Swann & Buhrmester, 2015; Swann et al., 2014). It is also possible that group members’ propensities to reveal personal and collective secrets are not independent. For example, stronger group identification produced by disclosing personal secrets may inhibit disclosure of collective secrets. Moreover, reduced disclosure of collective secrets, due perhaps to increased intergroup competition, may stimulate stronger group identification and thereby increased disclosure of personal secrets.

## Conclusions

The conditions under which group members reveal personal secrets to one another have not been systematically investigated by social psychologists. In a set of studies designed to clarify these conditions, we used Fiske’s Relational Models theory to generate hypotheses about when and why secret disclosure occurs. Our findings provided new information about how secrecy operates in group contexts, revealed yet another application of Fiske’s generative analysis of human sociality, and suggested a number of interesting questions for future research.

## Supplemental Material

sj-docx-1-gpi-10.1177_13684302231187870 – Supplemental material for Secret disclosure and social relationships in groups Supplemental material, sj-docx-1-gpi-10.1177_13684302231187870 for Secret disclosure and social relationships in groups by Giovanni A. Travaglino, John M. Levine, Dominik-Borna Ćepulić and Zhuo Li in Group Processes & Intergroup Relations

## Notes

1. In all studies, we report all conditions and measures unless otherwise noted. The Codebook and anonymized data are available at the following link: https://osf.io/cs2z4/

2. Participants also completed an unrelated measure of cultural values of horizontal, vertical individualism, and collectivism as a pilot for the translation of the scale.

3. Given prior findings, Authority Ranking and Market Pricing groups were not included.

4. Participants also completed additional items about the target group designed to mask our interest in the item assessing the total number of members in the group. The latter item was needed to calculate our criterion variable, namely the proportion of group members with whom participants shared secrets. See Appendix Codebook, Study 3, for a full list of items.

5. Asking participants to respond twice to all 38 secrets in Slepian et al.’s (2017) typology was deemed too demanding.

6. In interpreting model effects, it is important to note the difference between Nakagawa’s *R*^2^_*marginal*_ and *R*^2^_*conditional*_ (Nakagawa et al., 2017), which suggests that random effects substantially contributed to the explanation of the proportion of group members to whom the secret was revealed.
