# Supplementary material for: Secret disclosure and social relationships in groups
Source: Group Process Intergroup Relat. 2023 Aug 21;27(4):946–66. doi: 10.1177/13684302231187870 (PMC11161326; doi:10.1177/13684302231187870)
Supplement: sj-docx-1-gpi-10.1177_13684302231187870 – Supplemental material for Secret disclosure and social relationships in groups [file sj-docx-1-gpi-10.1177_13684302231187870.docx]

**Secret Disclosure and Social Relationships in Groups**

Appendix

| Table A. Fixed and random effects of the generalized linear mixed model reported in Study 3 with all reciprocity items included. | | | | | | | |
| --- | --- | --- | --- | --- | --- | --- | --- |
| **Effect** | **Parameter** | | ***b*** | ***SE*** | **95% CI** | ***z*** | ***p*** |
|  | (Intercept) | | -1.66 | 0.15 | [-1.96, -1.37] | -11.01 | < .001 |
| Fixed | group [EM] | | -0.22 | 0.06 | [-0.33, -0.11] | -3.87 | < .001 |
|  |  | (Intercept) [CS] | -1.55 | 0.15 | [-1.85, -1.25] | -10.10 | < .001 |
|  |  | (Intercept) [EM] | -1.77 | 0.15 | [-2.07, -1.47] | -11.55 | < .001 |
|  | reciprocity_1 | | 0.07 | 0.04 | [0.00, 0.14] | 2.03 | 0.043 |
|  | reciprocity_1 × group [EM] | | -0.03 | 0.06 | [-0.15, 0.09] | -0.49 | 0.624 |
|  |  | reciprocity_1 [CS] | 0.09 | 0.05 | [-0.01, 0.18] | 1.81 | 0.071 |
|  |  | reciprocity_1 [EM] | 0.06 | 0.05 | [-0.03, 0.15] | 1.24 | 0.213 |
|  | reciprocity_2 | | -0.03 | 0.03 | [-0.09, 0.03] | -0.96 | 0.338 |
|  | reciprocity_2 × group [EM] | | 0.02 | 0.04 | [-0.06, 0.10] | 0.51 | 0.609 |
|  |  | reciprocity_2 [CS] | -0.04 | 0.03 | [-0.11, 0.03] | -1.20 | 0.232 |
|  |  | reciprocity_2 [EM] | -0.02 | 0.04 | [-0.09, 0.06] | -0.46 | 0.644 |
|  | reciprocity_3 | | 0.03 | 0.03 | [-0.02, 0.09] | 1.29 | 0.199 |
|  | reciprocity_3 × group [EM] | | -0.09 | 0.04 | [-0.16, -0.02] | -2.40 | 0.016 |
|  |  | reciprocity_3 [CS] | 0.08 | 0.03 | [ 0.02, 0.13] | 2.62 | 0.009 |
|  |  | reciprocity_3 [EM] | -0.01 | 0.03 | [-0.08, 0.06] | -0.28 | 0.777 |
|  | reciprocity_4 | | -0.05 | 0.03 | [-0.11, 0.00] | -1.89 | 0.059 |
|  | reciprocity_4 × group [EM] | | 0.13 | 0.04 | [ 0.04, 0.21] | 2.89 | 0.004 |
|  |  | reciprocity_4 [CS] | -0.12 | 0.04 | [-0.19, -0.04] | -3.12 | 0.002 |
|  |  | reciprocity_4 [EM] | 0.01 | 0.03 | [-0.06, 0.08] | 0.29 | 0.774 |
|  | identity fusion | | 0.30 | 0.04 | [0.22, 0.39] | 6.89 | < .001 |
|  | identity fusion × group [EM] | | 0.19 | 0.06 | [ 0.06, 0.32] | 2.92 | 0.003 |
|  |  | identity fusion [CS] | 0.21 | 0.06 | [ 0.10, 0.32] | 3.67 | < .001 |
|  |  | identity fusion [EM] | 0.40 | 0.05 | [0.29, 0.50] | 7.64 | < .001 |
| Random | *Var*(secret type) | | 0.226 |  |  |  |  |
|  | *Var*(subject) | | 0.990 |  |  |  |  |
| Nakagawa's *R^2^_marginal_ / R^2^_conditional_* | | | 0.058 / 0.386^*^ | |  |  |  |

Note. Overdispersion parameter for the beta-binomial family was 1.55. The outcome variable is expressed as the logit of proportion of group members with whom the secret was shared. Indentations show simple effects. Communal sharing is coded with 0 and equality matching with 1 in the group variable. Reciprocity items were 1) “If I help other group members, they help me in return”, 2) “I feel obligated to help others who help me”, 3) “Members who don't reciprocate favors are violating group norms”, 4) “The rule in this group is, I won't help you unless you help me.

* Because it was not possible to estimate the R^2^ values for the beta-binomial model using Ludeke et al. (2020)’ package, we used estimates from the original binomial model..

Figure A. Marginal effects of reciprocity in communal sharing (CS) and equality matching (EM) groups, with the other predictors fixed at its mean. The grey area around lines represents the 95% confidence interval of the parameters


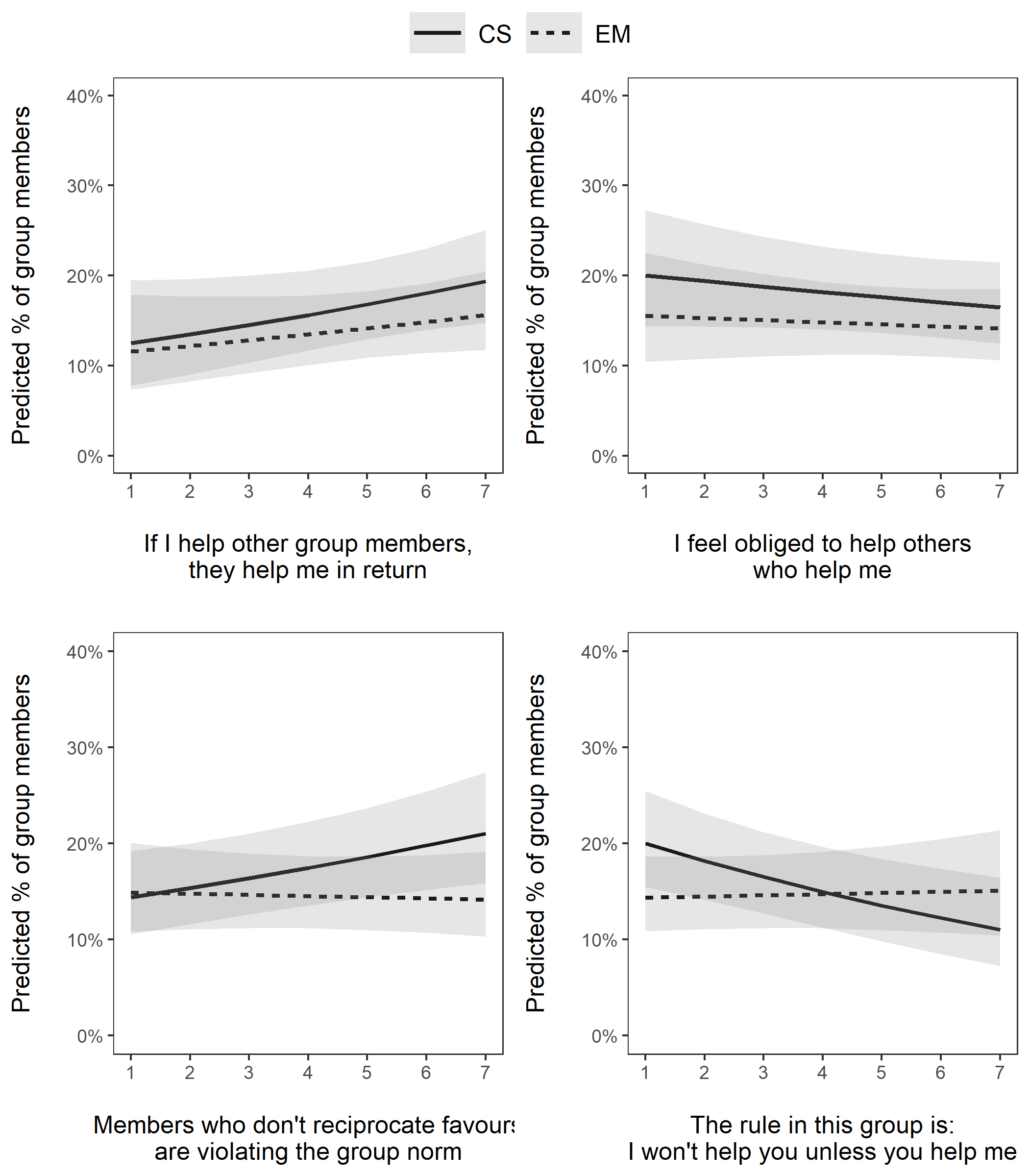


Figure B. Marginal effects of identity fusion in communal sharing (CS) and equality matching (EM) groups, with the other predictors fixed at its mean. The grey area around lines represents the 95% confidence interval of the paramters


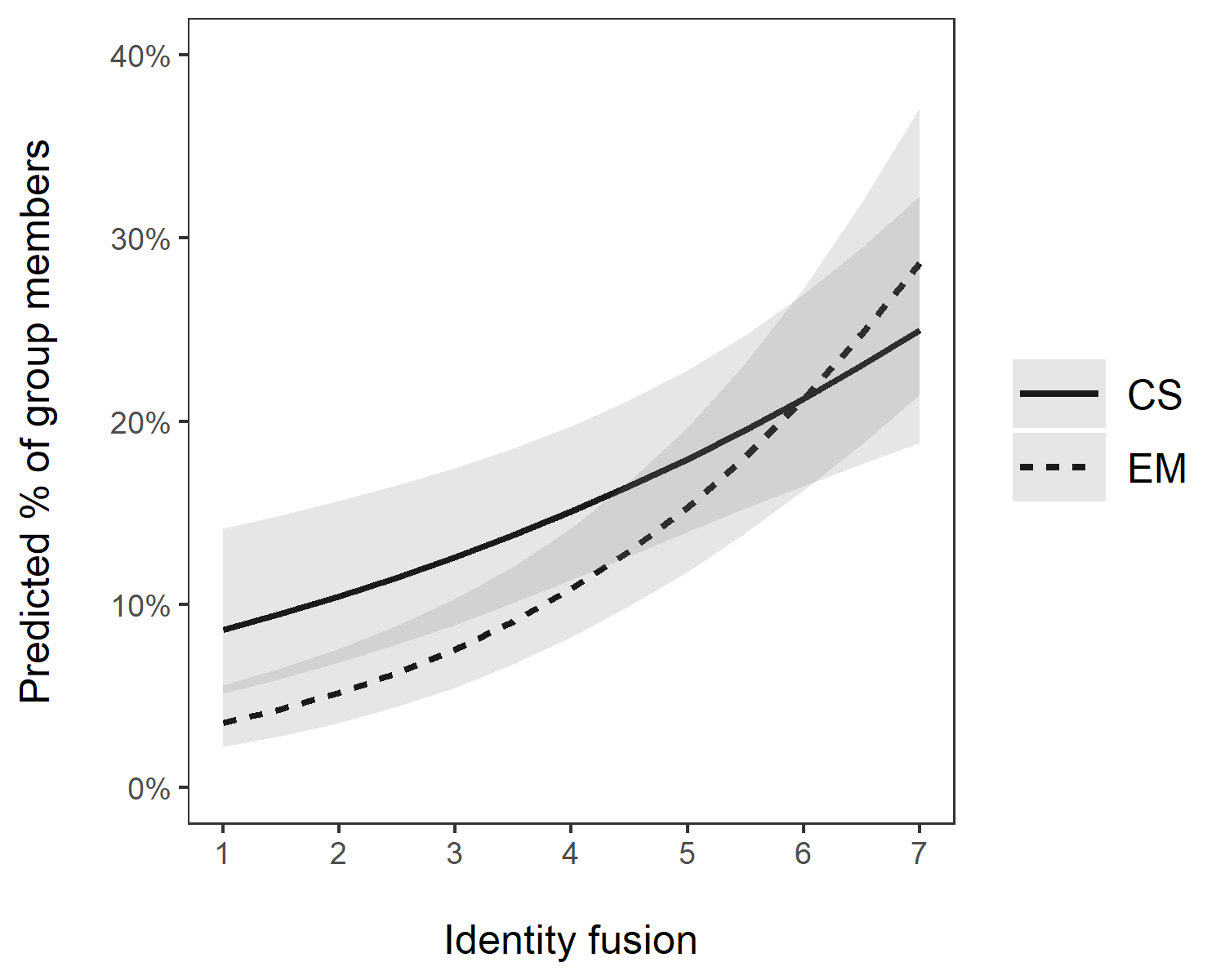


Figure C. Distribution of observed means transformed to the predicted percentages of group members to whom different secret types were revealed. Reciprocity and identification are fixed at their means, whereas the subject-level random effects are held at their population level.

**
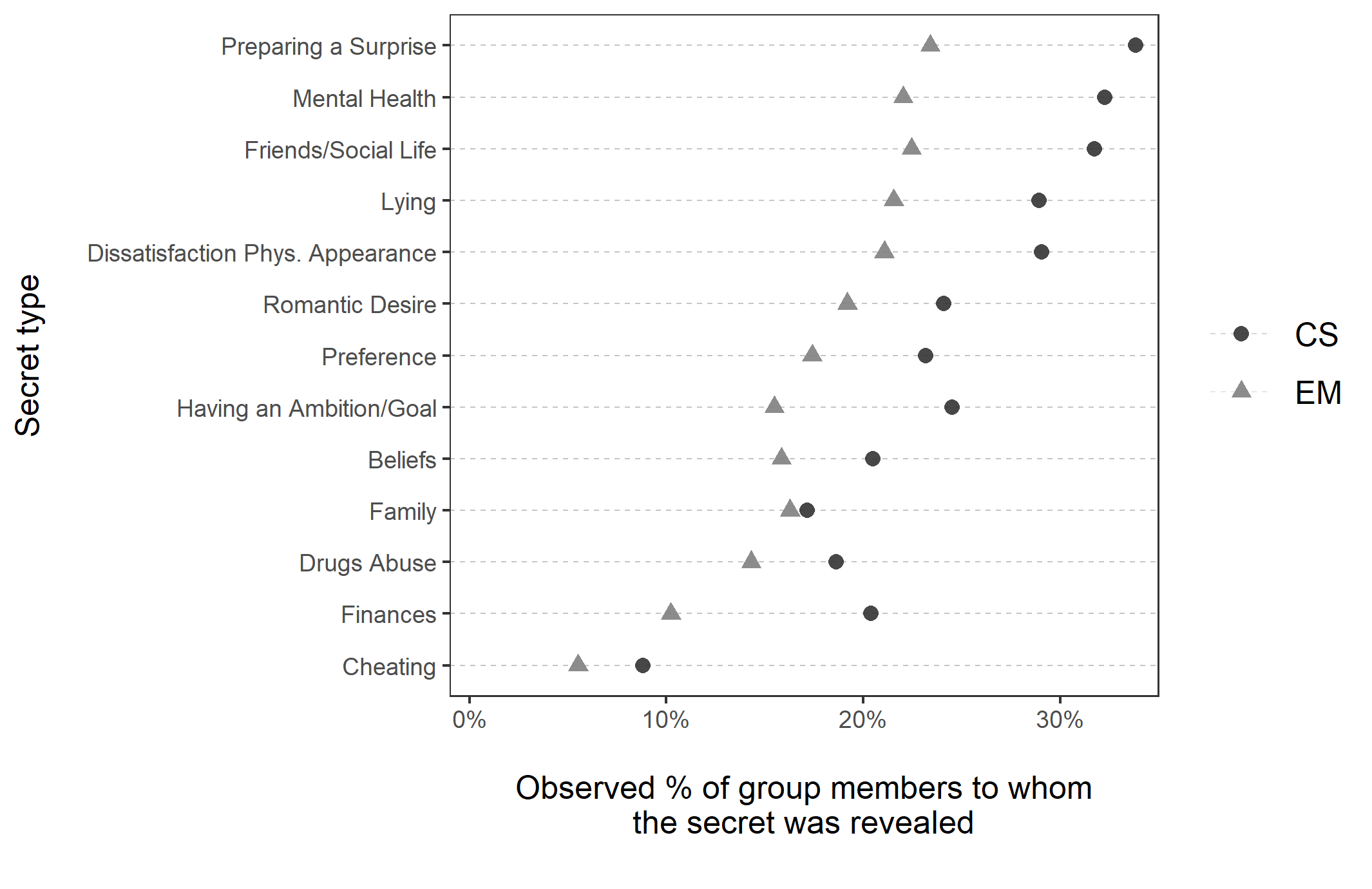
**

*Note.* The categories of Secrets were adapted from Slepian et al. (2017). *Surprise*: “Planning a surprise for someone (other than a marriage proposal)”; *Mental Health*: “Had mental health issues, or are dissatisfied with something about yourself other than physical appearance (such as fears, anxieties, depression, mental disorders, eating disorders)”; *Friends/Social Life*: “Dislike a friend, or are unhappy with your current social life”; *Lying*: “Have lied to someone”; *Dissatisfaction Phys. Appearance*: “Dissatisfied with your physical appearance”; *Romantic Desire*: “Had romantic desires about someone (while being single). For example, a crush, being in love with someone, wanting relations with a specific person (while being single)”; *Preference*: “Kept secret a preference for something?”; *Having an Ambition/Goal*: “Kept a secret ambition, secret plan, or secret goal for yourself?”; *Beliefs*: “Kept a belief secret? (for example, political views, religious views, views about social groups, prejudice)”; *Family*: “Kept a detail about your family secret?” *Drug Abuse*: “Used illegal drugs, or abused/addicted to a legal drug (e.g., alcohol, painkillers)”; *Finances*: “Kept secret details about finances (or amount of money you have)?”; *Cheating*: “Thought about having sexual relations with another person (while already in a relationship).
